# Inadequate maternal weight gain in the third trimester increases the risk of intrauterine growth restriction in rural Bangladesh

**DOI:** 10.1371/journal.pone.0212116

**Published:** 2019-02-08

**Authors:** S. M. Tafsir Hasan, Md. Alfazal Khan, Tahmeed Ahmed

**Affiliations:** Nutrition and Clinical Services Division, icddr,b, Dhaka, Bangladesh; Burnet Institute, AUSTRALIA

## Abstract

**Objective:**

To estimate the effect of inadequate maternal weight gain in the third trimester on the risk of intrauterine growth restriction (IUGR) in rural Bangladesh.

**Methods:**

This study analyzed data from 1,463 mother-infant pairs in Matlab, Bangladesh which were available through the electronic databases of Matlab Health and Demographic Surveillance System and Matlab hospital of the International Centre for Diarrhoeal Disease Research, Bangladesh (icddr,b). All the mothers were admitted to Matlab hospital for childbirth from January 2012 to December 2014, and they had singleton live births at term. Third-trimester weight gain (kg) was calculated by subtracting the estimated weight at the end of the second trimester from the weight taken before childbirth. Inadequate third-trimester weight gain was defined as 4 kg or less irrespective of pre-gravid nutritional status. IUGR was defined as a birth weight below 2500 g in full-term newborns (LBW-Term), and a birth weight for gestational age and infant sex less than the 10^th^ percentile (SGA-10^th^) and 2 standard deviations below the mean birth weight (SGA-2SD) based on the international newborn standards from the INTERGROWTH-21^st^ project. Multivariable logistic regression models were fitted to determine the independent effect of inadequate weight gain in the third trimester on the risk of IUGR.

**Results:**

A total of 824 (56.3%) women experienced inadequate weight gain in the third trimester of pregnancy. In this study, 215 (14.7%), 573 (39.2%) and 220 (15.0%) infants were born as LBW-Term, SGA-10^th^ and SGA-2SD, respectively. In the multivariable logistic regression models, compared to adequate weight gain in the third-trimester, the odds ratios (OR) for LBW-Term, SGA-10^th^ and SGA-2SD for inadequate weight gain were 1.8 (95% CI: 1.3, 2.5; p < 0.001), 1.4 (95% CI: 1.1, 1.8; p = 0.002) and 1.8 (95% CI: 1.3, 2.4; p = 0.001), respectively.

**Conclusions:**

Both inadequate third-trimester weight gain and IUGR are prevailing public health concerns in rural Bangladesh. Inadequate weight gain in the third trimester substantially increased the risk of IUGR. Public health programs focusing on the promotion of adequate weight gain in the third trimester of pregnancy with an ultimate aim to decrease IUGR should be implemented.

## Introduction

Intrauterine growth restriction (IUGR), a process of reduced fetal growth velocity failing the fetus to attain its growth potential, is associated with an increased risk of childhood mortality and morbidity, cognitive impairment, higher incidence of chronic diseases in adulthood and overall reduced human capital [f1–4]. In low and middle income countries (LMICs), each year 23.3 million infants are estimated to be born small for gestational age (SGA) indicating IUGR. Bangladesh ranks fourth among the countries with the highest burden of IUGR with an SGA prevalence of 30.5% [5]. The modifiable risk factors for IUGR range from poor maternal nutrition to maternal infections, adolescent pregnancy, short birth spacing, smoking, etc. [5, 6]. Studies of gestational weight gain (GWG) also demonstrate an elevated risk of IUGR in women having inadequate weight gain during pregnancy [2]. However, suboptimal weight gain in the early, middle and late pregnancy affects fetal growth differently [7, 8]. Weight gain in the first trimester of pregnancy has generally been found to be unrelated to birth weight, probably because of the minimal fetal growth during this period [9–11]. Several epidemiologic studies have reported a strong association of second-trimester weight gain with birth weight [11–14]. Although the fetus grows most rapidly during the third trimester [11, 12], literature varies for the effect of third-trimester weight gain on birth outcomes. For example, Siega-riz et al. have shown that an inadequate rate of weight gain in the third trimester increases the risk of preterm birth [15]. Strauss and Dietz have reported an approximately two-fold increase in the risk of IUGR with low maternal weight gain in either the second or the third trimester [11]. On the contrary, Drehmer et al. present evidence that suboptimal maternal weight gain in the second but not in the third trimester is associated with an elevated risk of SGA [16]. However, contemporary studies examining the association of adequacy of third-trimester weight gain with fetal growth are scarce.

Maternal undernutrition and inadequate GWG are rife in rural communities of LMICs despite the emergence of the global obesity epidemic [17–19]. In addition, women in rural Bangladesh usually seek facility-based prenatal care in the late second or early third trimester [20]. This behavior is also common among women elsewhere in LMICs giving only during the third trimester a realistic chance to intervene in these marginally nourished populations [21–23]. Furthermore, much of the available research on the topic comes from high-income countries with well-nourished populations with a focus on overweight/obesity [24] which warrants further investigations in LMICs as the pattern and influence of GWG can vary depending on maternal ethnicity, anthropometry, and context [2, 25]. This study thus aimed to estimate the effect of inadequate maternal weight gain in the third trimester on the risk of IUGR in rural Bangladesh.

## Materials and methods

### Study population and data source

Data on GWG and potential confounders were derived from the Pregnancy Weight Gain study which primarily aimed to estimate the prevalence and risk factors of inadequate weight gain in the third trimester in rural Bangladesh. It was conducted in Matlab, Bangladesh where the International Centre for Diarrhoeal Disease Research, Bangladesh (icddr,b) has been running for 50 years a prospective Health and Demographic Surveillance System (HDSS) comprising a population of 230,000 [20]. Details of the study methodology have been reported previously [26]. In brief, the study included 1,883 pregnant women who were admitted to Matlab hospital of icddr,b for childbirth from January 2012 to December 2014. These women had singleton live births at term, and they had visited the facility for a prenatal check-up during the late second trimester (26.1 ± 1.4 weeks). Gestational age was based on the date of the last menstrual period (LMP) and confirmed by ultrasonographic estimation in the second trimester of pregnancy. Pregnant women were weighed to the nearest 100 g at Matlab hospital during the second-trimester visit and before childbirth by trained nurses using Tanita HD-661 digital weighing scales. Women with documented major illnesses, preexisting or found during the prenatal check-up, or unavailability of information on weight gain were not considered in the study. In the present study, we kept the analyses limited to a sub-sample of 1,463 mother-infant pairs whose delivery was conducted from 37 completed to 42 weeks of gestation and infant weight was measured within 72 hours of birth. A total of 420 cases were left out (415 due to weighing of infants after 72 hours of birth and 5 due to childbirth after 42 weeks of gestation) from the original dataset. Data on birth weight were available through the electronic database of Matlab hospital. Trained nurses conducted the weight measurements of the newborns at Matlab hospital using a Tanita-1584 Baby Scale (digital weighing scale) with 20 g sensitivity.

### Third-trimester weight gain

This study defined third-trimester weight gain as the weight gained by a woman from the beginning of the 29^th^ week of gestation until childbirth [27–29].

For each woman, the weekly rate of weight gain (kg/week) during the third trimester was estimated using the following equation:

weekly rate of weight gain = (last weight before childbirth ─ weight at prenatal checkup during the late second trimester) ÷ (gestational age when last weight measured ─ gestational age at prenatal checkup during the late second trimester)

The total third-trimester weight gain (kg) for each woman was interpolated using the following equation:

total third trimester weight gain = last weight before childbirth ─ [weight at prenatal checkup during the late second trimester + (28 ─ gestational age at prenatal checkup during the late second trimester) × weekly rate of weight gain]

The adequacy of third-trimester weight gain was assessed based on the recommendation made for Bangladeshi women by Ahmed et al. [17]. Women were categorized as having inadequate weight gain in the third trimester if they gained 4 kg or less during this period, an amount that was considered insufficient regardless of pre-pregnancy nutritional status. Gaining more than 4 kg was deemed to be adequate.

### Covariates

Following maternal and infant characteristics were considered as covariates: maternal age (≤19 years, 20–34 years, or ≥35 years) [11], height (≤145 cm, 146–155 cm, or >155 cm) [30], parity (nulliparous or parous) [31], level of education (≤5 years, 6–9 years, or ≥10 years) [26], socioeconomic status (wealth quintile) [32], infant sex (male or female) and gestational age at birth (duration of pregnancy) [33]. Wealth quintile, an indicator of household-level wealth consistent with expenditure and income measures, was computed by HDSS using household asset data via principal component analysis [32]. Wealth quintile had some (5.9%) missing values in the original dataset which have been replaced by real values by revisiting the updated HDSS database. However, when examined, neither the missingness was associated with any study outcome, nor the estimates in the regression models changed considerably after replacing the missing values with real ones.

### IUGR

We used three proxy measures to define IUGR, such as LBW-Term, SGA-10^th^ and SGA-2SD. LBW-Term was defined as a birth weight below 2500 g of an infant born after 37 completed weeks of gestation. SGA-10^th^ was defined as a birth weight less than the 10^th^ percentile of birth weight by sex for a specific gestational age of the reference population. SGA-2SD was defined as a birth weight 2 standard deviations below the mean birth weight for a specific gestational age and infant sex of the reference population. We used the international newborn standards from the INTERGROWTH-21^st^ project as the reference population [33].

We chose to examine LBW-Term because it is a convenient way to define IUGR, has been used in previous studies [11, 18], and found to be associated with increased infant morbidity and mortality and long-term health consequences [3, 34]. SGA-10^th^ is the preferred definition of small for gestational age by a World Health Organization (WHO) Expert Committee [35] and the American College of Obstetrics and Gynecology [36] and is a commonly used proxy measure of IUGR [5]. We examined a more restrictive proxy measure of IUGR, SGA-2SD because it has been shown to best identify infants at risk of morbidity and mortality [37, 38].

### Statistical analysis

We found the continuous variables of interest to be normal or visually close to normal, and did not consider any transformations (data not shown). We compared the background characteristics of the participants in the present sample with those who were excluded using Student’s t-test for continuous variables and chi-square test for categorical variables. We presented the sociodemographic, anthropometric, pregnancy and birth outcome related characteristics of mothers and infants in the sample as mean ± standard deviation for continuous variables and frequency measures for categorical variables. We visualized the bivariate relationship between infant birth weight and maternal rate of weight gain in the third trimester through a scatterplot with superimposed locally weighted and fitted regression lines. We used simple logistic regression to examine the bivariate association between third-trimester weight gain and LBW-Term, SGA-10^th^, and SGA-2SD. We fitted three separate multivariable binary logistic regression models to estimate the independent effect of inadequate maternal third-trimester weight gain on the risk of LBW-Term, SGA-10^th^, and SGA-2SD. We calculated odds ratios with 95% confidence intervals (95% CI) by using adequate weight gain in the third trimester as the referent. Additionally, we assessed the association of infant birth weight with maternal rate of weight gain in the third trimester using simple and multiple linear regression. In the multivariable models, all the covariates of a priori interest were included. Statistical significance was set at p < 0.05. An MS Windows-based application entitled “Neonatal Size Calculator for newborn infants between 24^+0^ and 42^+6^ weeks' gestation” available on the INTERGROWTH-21^st^ website (https://intergrowth21.tghn.org) was used to calculate the percentiles and Z scores of birth weight specific for gestational age and infant sex. All other statistical analyses were performed with Stata/PC (StataCorp, College Station, Texas 77845 USA, version 14.1).

### Ethics statement

This study used de-identified routinely collected data which were available through the electronic databases of Matlab HDSS and Matlab hospital. The study did not involve any interviews with the participants. The study protocol (PR-16028) was reviewed and approved by the icddr,b research and ethical review committees (Institutional Review Board of icddr,b).

## Results

No meaningful differences were observed with regard to background characteristics between the participants included and excluded from the analysis except nulliparity. The proportion of nulliparous women were lower in the present sample (41.8% vs. 51.0%) (S1 Table).

In our study sample of 1,463 mother-infant dyads, 20.6% of the mothers were adolescents, and the mean maternal age was 24.5 ± 5.6 years. About fifteen percent of the women were short-statured (≤145 cm), and 41.8% were nulliparous. One-fifth of the women (20.6%) had the education of 10 years or more, and about half belonged to families in the fourth (21.1%) or the highest (26.8%) wealth quintiles (Table 1).

**Table 1:** Sociodemographic, anthropometric, pregnancy and birth outcome related characteristics of mothers and infants (n = 1463).

Maternal age (years)	
≤19, n (%)	302 (20.6)
20–34, n (%)	1071 (73.2)
≥35, n (%)	90 (6.2)
Mean ± SD	24.5 ± 5.6
Maternal height (cm)	
Short (≤145), n (%)	218 (14.9)
Average (146–155), n (%)	965 (66.0)
Tall (>155), n (%)	280 (19.1)
Mean ± SD	151.0 ± 5.3
Parity	
Nulliparous, n (%)	612 (41.8)
Parous, n (%)	851 (58.2)
Duration of pregnancy (weeks), Mean ± SD	39.2 ± 1.1
Maternal education (years)	
≤5, n (%)	334 (22.8)
6–9, n (%)	827 (56.5)
≥10, n (%)	302 (20.6)
Mean ± SD	7.5 ± 2.9
Wealth quintile	
Lowest, n (%)	234 (16.0)
Second, n (%)	245 (16.8)
Middle, n (%)	284 (19.4)
Fourth, n (%)	308 (21.1)
Highest, n (%)	392 (26.8)
Rate of weight gain (kg/week), Mean ± SD	0.33 ± 0.2
Third trimester weight gain (kg)	
Inadequate (≤4)	824 (56.3)
adequate (>4)	639 (43.7)
Mean ± SD	3.7 ± 2.2
Infant sex	
Male, n (%)	737 (50.4)
Female, n (%)	726 (49.6)
Birth weight (g), Mean ± SD	2874.6 ± 416.9
LBW-Term, n (%)	215 (14.7)
SGA-10^th^, n (%)	573 (39.2)
SGA-2SD, n (%)	220 (15.0)

SD, Standard deviation.

Of the women included in the study, 56.3% of the women experienced inadequate weight gain in the third trimester of pregnancy. The mean rate of weight gain and total weight gain in the third trimester were 0.33 ± 0.2 kg/week and 3.7 ± 2.2 kg, respectively. In the present sample, the mean birth weight was 2874.6 ± 416.9 g. The prevalence of LBW-Term, SGA-10^th^ and SGA-2SD were 14.7%, 39.2% and 15%, respectively (Table 1).

**Fig 1:**
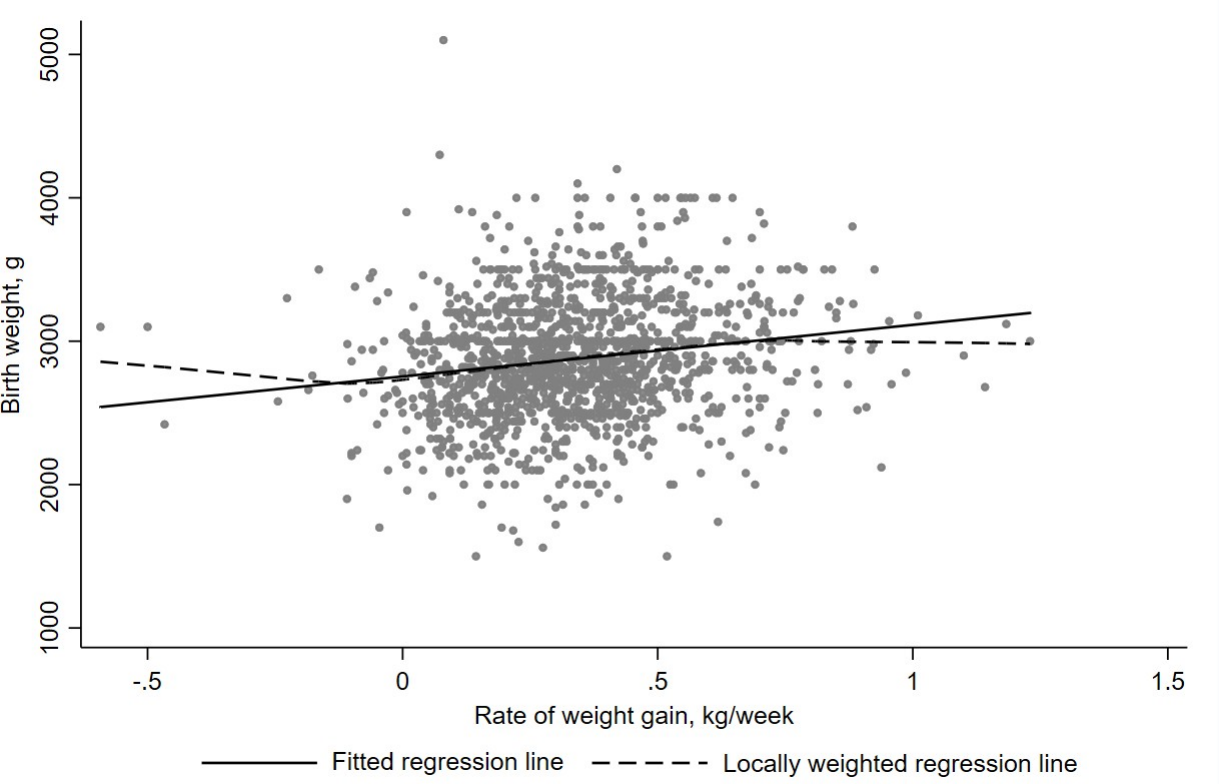
Regression (lines) of infant birth weight on maternal rate of weight gain in the third trimester (n = 1463).

Rate of weight gain in the third trimester appeared to have a positive and approximately linear association with infant birth weight (Fig 1).

In the adjusted model, each unit increase in the rate of weight gain (kg/week) during the third trimester was associated with a 326.7 g (95% CI: 224.0, 429.4; p < 0.001) increase in infant birth weight (Table 2).

**Table 2:** Association between maternal rate of weight gain in the third trimester and infant birth weight using linear regression (n = 1463).

	Birth weight (g)			
	β (95% CI) [Unadjusted]	*p* value	β (95% CI) [Adjusted]^a^	*p* value
**Rate of weight gain (kg/week)**	360.0 (254.5, 465.4)	<0.001	326.7 (224.0, 429.4)	<0.001

^a^Adjusted for maternal age, height, parity, education, wealth quintile, infant sex and gestational age at birth.

Table 3 demonstrates the adjusted and unadjusted results of the logistic regression models evaluating IUGR as a function of inadequate third-trimester weight gain. Simple logistic regression analysis showed that inadequate maternal third-trimester weight gain was significantly associated with LBW-Term (p < 0.001), SGA-10^th^ (p < 0.001) and SGA-2SD (p < 0.001). In the multivariable logistic regression models, compared to adequate weight gain in the third-trimester, the odds ratios (OR) for LBW-Term, SGA-10^th^ and SGA-2SD for inadequate weight gain were 1.8 (95% CI: 1.3, 2.5; p < 0.001), 1.4 (95% CI: 1.1, 1.8; p = 0.002), and 1.8 (95% CI: 1.3, 2.4; p = 0.001), respectively.

**Table 3:** Risk of IUGR based on inadequate maternal weight gain in the third trimester by logistic regression (n = 1463).

	OR (95% CI) [Crude]	*p* value	OR (95% CI) [Adjusted]	*p* value
**LBW-Term**	1.9 (1.4, 2.6)	<0.001	1.8 (1.3, 2.5)^a^	<0.001
**SGA-10**^**th**^	1.5 (1.2, 1.9)	<0.001	1.4 (1.1, 1.8)^b^	0.002
**SGA-2SD**	1.9 (1.4, 2.6)	<0.001	1.8 (1.3, 2.4)^b^	0.001

^a^Adjusted for maternal age, height, parity, education, wealth quintile and infant sex.

^b^Adjusted for maternal age, height, parity, education and wealth quintile.

## Discussion

We explored the association of third-trimester weight gain with fetal growth in a rural setting of a mother and infant pairs residing in Matlab, Bangladesh. In this study, inclusive of women with a high prevalence of short stature and adolescent pregnancy, more than half of the women experienced inadequate weight gain in the third trimester, and two-fifths of the infants were born with IUGR defined as birth weight less than the 10^th^ centile. This finding is consistent with the widespread maternal and child undernutrition reported elsewhere in LMICs [5, 19, 39].

Our study revealed that inadequate weight gain in the third trimester increased the risk of IUGR. This was further supported by the finding that rate of weight gain in the third trimester was positively associated with infant birth weight. During the third trimester of pregnancy, the placenta becomes most active in shunting nutrients to the fetus to keep up with the rapid fetal growth and fat accretion [40, 41]. Therefore, the relationship found in our study is biologically plausible. However, the effect of inadequate weight gain was variable across the definitions of IUGR; suboptimal third-trimester weight gain approximately doubled the risk of restricted fetal growth when it was defined as LBW-Term or SGA-2SD, but the effect seemed modest when defined as SGA-10^th^. To interpret this variability, it is essential to understand the strength and limitations of these definitions and their clinical relevance. Ideally, IUGR is a prenatal diagnosis based on serial ultrasound measurements during pregnancy [4]. Unfortunately, in developing countries for most pregnancies, it may not be possible to determine whether and when intrauterine growth is retarded by doing multiple ultrasound measurements in a longitudinal manner [42]. Instead, proxy measures are used to define IUGR. However, none of these are without limitations. LBW-Term underestimates the number of IUGR as it considers all the infants with a birth weight of 2500 g or above to be normal regardless of gestational age at birth. SGA-10^th^ includes some infants who are constitutionally small but healthy, thus may overestimate the prevalence of IUGR. SGA-2SD, although may be subject to higher false negatives, is the best indicator for identifying infants whose fetal growth is genuinely compromised [37, 38]. The inclusion of relatively common measures of IUGR such as LBW-Term and SGA-10^th^ allowed us to compare our findings with that of the similar studies, but we recommend that SGA-2SD should be given priority in gaging the effect of inadequate weight gain on IUGR.

Although previous studies have examined the association between gestational weight gain and fetal growth, few studies have specifically focused on the third trimester. Furthermore, there is a dearth of good-quality recent work, particularly in developing countries where it is relevant. To the best of our knowledge, ours is the first study in Bangladesh to comprehensively evaluate the association of fetal growth with maternal weight gain in the third trimester until when many women do not seek prenatal care in LMICs. In agreement with our findings, Strauss and Dietz previously showed that, across the spectrum of maternal pre-gravid nutritional status, low weight gain in the third trimester approximately doubled the risk of LBW-Term in American populations [11]. A relatively recent study from Papua New Guinea reported that a failure to gain weight for more than three weeks preceding childbirth increased the risk of several perinatal complications including LBW-Term (OR: 2.88, 95% CI: 1.83, 4.75) [18]. More recently, one standard deviation increase in any-prior-weight-independent weight gain during ≥30 weeks among Vietnamese women was found to be associated with a 38.9 g (95% CI: 14.7, 63.1) increase in birth weight and a 22% reduction in the risk of SGA-10^th^ (OR: 0.78, 95% CI: 0.63, 0.97) [43]. Similarly, Margerison-Zilko et al. showed that one kilogram increase in third-trimester weight gain was associated with a 10% reduction in the risk of SGA-10^th^ (OR: 0.90, 95% CI: 0.86, 0.94) [44]. Another study showed that maternal rate of weight gain from the 27^th^ week onward was independently associated with both fat mass and fat-free mass in newborns [45]. Unlike our study, Cho et al. did not find any evidence for the association of weight gain in late pregnancy with SGA-10^th^ among the Korean population. However, instead of examining weight gain per trimester, Cho et al. analyzed weight gain rates for three vaguely defined periods making no mention of specific gestational age [46].

The results of our study substantiate the necessity of adequate maternal weight gain in the third trimester. Although it is somewhat speculative, the high prevalence of inadequate weight gain and IUGR in this rural population may be attributed to inadequate dietary intake, lack of dietary diversity, limited knowledge about a balanced diet in pregnancy, poor access to high-quality health care, structural inequality, and strong cultural norms [26, 47–49]. Institute of Medicine (IOM) guidelines have stressed the importance of prenatal counseling to help women gain weight appropriately [2]. The counseling should be contextualized and culturally acceptable, and the emphasis should be on a balanced diet and regular weight monitoring. For a positive pregnancy experience, WHO recommends an early initiation of antenatal care [50]. However, since many women in LMICs do not seek prenatal care until the late second trimester, some alternative, innovative approach can be adopted. For women coming late for prenatal check-up, the provision of short term health education during the third trimester focusing on consuming an adequate and balanced diet may be a practicable solution [51]. If poverty and non-affordability of nutritious food are issues, then we need to think about developing a ready-to-use supplementary food to be consumed during pregnancy for targeted supplementation. Surprisingly, a recent systematic review found that increment in energy intake during pregnancy was not associated with gestational weight gain among well-nourished/overweight women predominantly living in developed countries [52]. However, it is not wise to generalize the effect of food supplementation across low-, middle- and high-income countries [53]. A recent Cochrane review presented evidence that prenatal education focusing on increasing energy and protein intake was able to increase birth weight among undernourished women. In addition, balanced energy and protein supplementation was found to improve fetal growth and reduce the risk of SGA in the general obstetric population [54]. Another review concluded that balanced protein energy supplementation in undernourished pregnant women in low- and middle-income countries significantly improved birth weight [55]. However, food supplementation in pregnancy to improve perinatal outcomes including gestational weight gain and fetal growth is too complicated an issue, and may only be understood through conduct of large, well-designed randomized controlled trials [54].

A number of well-designed and prospective studies, particularly from developed countries, preferably used and validated North American population-centered IOM guidelines on GWG [2, 45, 56–58]. In a recent systematic review and meta-analysis, Goldstein et al. showed that IOM guidelines on GWG might apply to women in the Western European and East Asian countries in addition to American women [59]. However, this review was unable to include studies from developing countries, and more importantly, from South Asia, which is ethnically and socio-culturally much different than East Asia, Europe or the USA. Furthermore, studies from India and Brazil presented evidence that IOM recommendations for optimal weight gain might not be appropriate for these populations [16, 60]. Also, IOM states that “… these guidelines are intended for use among women in the United States. They may be applicable to women in other developed countries. However, they are not intended for use in areas of the world where women are substantially shorter or thinner than American women or where adequate obstetric services are unavailable [2].” It is acknowledged that the definition of optimal gestational weight gain should be population-specific, because of differences in body built, socio-cultural context and nutritional status [61]. Therefore, in the present study, we assessed the adequacy of third-trimester weight gain based on the cut-off recommended for Bangladeshi women by Ahmed et al in the National Nutrition Programme (NNP) Baseline Survey 2004 report [17], which is an apt criterion for the marginally nourished and impoverished rural women lacking information on pre-gravid nutritional status [26]. The investigators of NNP Baseline Survey 2004, many of whom were national experts in maternal and child nutrition, comprehensively reviewed available information on GWG and its association with perinatal outcomes to decide upon an easy-to-use cut-off for weight gain to be recommended for Bangladeshi women through a face-to-face consensus meeting. Ahmed et al. proposed that Bangladeshi women should gain at least 9 kg throughout the pregnancy and more than 4 kg during the third trimester for full term pregnancies in order to reduce the risk of delivering low birth weight infants and other perinatal complications. They took into consideration, while reaching consensus, the evidence available from developing countries including from South Asia [62–67], the fact that weight gain is minimal in the first trimester (~1 kg) and approximately uniform in the second and third trimesters for full term pregnancies [68], and last but not least, the short stature, unique body built and socio-cultural context of Bangladeshi women (personal communication; Dr Tahmeed Ahmed, the lead investigator of NNP Baseline Survey 2004).

We had a large sample of mother and infant pairs with the availability of socioeconomic, demographic, and nutrition data to examine the independent association between third-trimester weight gain and fetal growth. However, our study is not without limitations. The retrospective nature of the data which were collected as a part of routine measures might have led to misclassification in this study. In addition, we were unable to classify women based on their pre-pregnancy nutritional status because this information was not available in the database. The present study included only full-term infants because preterm pregnancy does not allow applying the third-trimester cut-off we used in the study, and it may be associated with unidentified underlying pathologies [69]. Therefore, our results apply only to women who carry infants to term. Additionally, we estimated the third-trimester weight gain based on the assumption that each woman gained weight at a steady rate from the time of prenatal check-up (the late second trimester) until childbirth (throughout the third trimester). Although, this assumption is based on strong evidence [2, 68], and the estimation of rate of weight gain as well as total weight gain in the third trimester was done for each woman on individual basis, these calculations might be subject to bias due to variable weight gain rate throughout the pregnancy. Finally, although our study sample has similitude to the general population of Matlab with regard to background characteristics [20, 32], the results may not be nationally representative. Similar studies in other regions of Bangladesh are needed to confirm our findings.

In conclusion, this study demonstrated that inadequate weight gain in the third trimester substantially increased the risk of IUGR. This issue is pertinent given the high prevalence of short maternal stature, adolescent pregnancy, inadequate weight gain, IUGR and generally low socioeconomic status in rural Bangladesh. Appropriate management of prenatal maternal nutrition is of utmost importance. Since many women in LMICs do not seek antenatal care services until the late second trimester, innovative public health programs to effectively improve weight gain during the third trimester should be explored and receive support.

## Supporting information

S1 Table Background characteristics of mothers and infants included and excluded from the study sample. (PDF)

S2 Table Percentages of inappropriate size at birth in the study sample (n = 1463). (PDF)
