# Supplementary material for: Inadequate maternal weight gain in the third trimester increases the risk of intrauterine growth restriction in rural Bangladesh
Source: PLoS One. 2019 Feb 8;14(2):e0212116. doi: 10.1371/journal.pone.0212116 (PMC6368315; doi:10.1371/journal.pone.0212116)
Supplement: S1 Table — (PDF) [file pone.0212116.s001.pdf]

**Characteristic**	**Study sample n = 1463**	**Excluded subjects n = 420**	***p* value**
Age (years)	24.5 ± 5.6	24.5 ± 6.1	0.844
Height (cm)	151.0 ± 5.3	150.7 ± 5.4	0.268
Nulliparity, n (%)	612 (41.8)	214 (51.0)	0.001
Duration of pregnancy (weeks)	39.2 ± 1.1	39.2 ± 1.3	0.364
Education (years)	7.5 ± 2.9	7.4 ± 3.2	0.582
Wealth quintile			
Lowest, n (%)	234 (16.0)	64 (15.2)	
Second, n (%)	245 (16.8)	78 (18.6)	
Middle, n (%)	284 (19.4)	73 (17.4)	0.801
Fourth, n (%)	308 (21.1)	93 (22.1)	
Highest, n (%)	392 (26.8)	112 (26.7)	
Infant sex: Male, n (%)	737 (50.4)	209 (49.8)	0.824

Values are mean ± SD unless otherwise specified.
