# Supplementary material for: Inadequate maternal weight gain in the third trimester increases the risk of intrauterine growth restriction in rural Bangladesh
Source: PLoS One. 2019 Feb 8;14(2):e0212116. doi: 10.1371/journal.pone.0212116 (PMC6368315; doi:10.1371/journal.pone.0212116)
Supplement: S2 Table — (PDF) [file pone.0212116.s002.pdf]

**Size at birth**	**n (%)**
Low birthweight (birth weight <2500 g)	215 (14.7)
Small for gestational age (birth weight <10th centile for GA and infant sex)	573 (39.2)
Small for gestational age (birth weight <-2 SDs for GA and infant sex)	220 (15.0)
Macrosomia (birth weight ≥4000 g)	21 (1.4)
Macrosomia (birth weight ≥4500 g)	1 (0.1)
Large for gestational age (birth weight >90th centile for GA and infant sex)	27 (1.9)
Large for gestational age (birth weight >2 SDs for GA and infant sex)	4 (0.3)

GA, gestational age; SD, standard deviation.

The international newborn standards from the INTERGROWTH-21st project were used as the reference to determine small/large for gestational age.
